# Barrier resolution via patient navigation in the context of cancer care

**DOI:** 10.1007/s00520-026-10413-7

**Published:** 2026-02-11

**Authors:** Elizabeth S. Ver Hoeve, Patrick Wightman, Elizabeth Calhoun, Monica Hernandez, Julie S. Armin, Heidi A. Hamann

**Affiliations:** 1 Department of Family Medicine and Community Health, University of Wisconsin-Madison, Madison, WI 53705 USA; 2 University of Arizona, Tucson, AZ 85721 USA; 3 Banner University Medical Center, Tucson, AZ 85724 USA; 4 University of Illinois at Chicago, Chicago, IL 60607 USA

**Keywords:** Barrier resolution, Patient navigation, Cancer, Program effectiveness

## Abstract

**Purpose:**

Patient navigation programs have demonstrated intervention efficacy associated with barrier reduction and health outcome improvements in the context of cancer care. Greater characterization of barriers and barrier resolution likelihoods may support program optimization.

**Methods:**

A 3-month longitudinal, non-comparative community-focused (i.e., lay navigator) patient navigation program was implemented at an NCI-designed cancer center between 2018 and 2021. Barriers to cancer care were reported by patients (*n* = 207) at pre-intervention and re-assessed at post-intervention. Descriptive analyses examined patient-level associations among pre-intervention barriers and post-intervention rates of barrier resolution. Logistic regressions were conducted at the barrier level and patient level to estimate the likelihood of barrier resolution associated with *Health Access*, *Financial*, and *Psychosocial* barrier domains.

**Results:**

Participants reported an average of 3.54 distinct barriers to cancer care. Barriers associated with *Health Access* and *Financial* domains were most frequently endorsed. Post-navigation, barriers were found to differ in their resolution rates. At the barrier domain level, resolution rates differed significantly (*X*^2^(2) = 7.826, *p* = 0.02), with *Financial* barriers significantly less likely (OR = 0.61; 95% CI = 0.41, 0.89) to be resolved. For participants who reported barriers exclusively within the *Financial* domain, the odds of any of their barriers being “resolved” were approximately 77% lower compared to participants who reported barriers in other domains.

**Conclusion:**

In this community-focused patient navigation program, barriers in the *Financial* domain proved to be the most difficult to resolve. The amenability of specific barriers to resolution via patient navigation can guide program tailoring and optimization.

## Introduction

Cancer health disparities vary by socioeconomic status, geography, race, ethnicity, and comorbidity burden [1]; manifest across the cancer care continuum [2]; and contribute to higher cancer incidence and likelihood of cancer-related mortality [3, 4]. Patient-reported barriers to cancer care serve as one of the primary drivers of cancer health disparities [5, 6], disproportionally impacting medically underserved patients and widening exiting disparities [3]. Patient navigation is one of the most well-studied and efficacious interventions for reducing patients’ barriers to cancer care [7–10], improving cancer care coordination [11], and enhancing patient health outcomes [12–19]. Patient navigation has been heralded as a particularly effective intervention among medically underserved patients with cancer [11, 12, 20–25], and was recently approved by the Centers for Medicare and Medicaid [26] as a reimbursable cancer care service. Yet, despite widespread implementation of patient navigation programs in cancer centers across the USA, national statistics on most cancer health disparities have remained relatively stagnant [12, 27, 28], indicating that further optimization of patient navigation program effectiveness is warranted.

Absence of standardized definitions for describing patients’ barriers to care as well as inconsistent criteria for evaluating efforts to address and resolve those barriers [29] may limit the ability of patient navigation programs to meaningfully impact cancer health disparities. The Patient Navigation Research Program (PNRP) developed best practice guidelines for documentation of patient-reported barriers to care as well as patient navigator actions to address and resolve those barriers [30], and several programs have explicitly incorporated PNRP guidance [31–34]. Utilization of consistent criteria for identifying, categorizing, and evaluating what it means to sufficiently “resolve” an individual patient’s barriers to cancer care [35] could provide a foundation for shared understanding as to which barriers are more or less amenable to resolution via patient navigation. Program optimization based on barrier resolution likelihoods could further allow patient navigation programs to more effectively prioritize resources, capitalize on navigators’ core competencies [36], and ameliorate cancer health disparities [37].

As part of a community-focused (i.e., lay) patient navigation intervention introduced at an NCI-designated comprehensive cancer center, a sample of primarily medically underserved patients with cancer reported on the discrete barriers to cancer care that they were experiencing prior to and following participation in a patient navigation intervention. The implementation effectiveness results of this intervention have been reported previously [10]. The aims of this paper are to explore patterns of barrier resolution through detailed (1) characterization and categorization of patient-reported barriers to cancer care at pre-intervention, (2) assessment of barrier resolution at the barrier level, and (3) assessment of barrier resolution at the patient level.

## Method

### Study design

A community-focused (i.e., lay) patient navigation program was introduced at an NCI-designated Cancer Center in the Southwestern United States and operated between June 2018 and August 2021. The intervention included a 3-month longitudinal non-comparative measurement period in which participants reported their barriers to cancer care at enrollment (i.e., pre-intervention) and those barriers were assessed for degree of resolution by trained research staff at 3months post-navigation. This study received ethical approval from the Institutional Review Board at the University of Arizona (#1804483104), and informed consent was obtained from all participants. Detailed information about the larger study design can be found in Ver Hoeve et al. (2024) [10].

### Assessments and measures

#### Demographics and cancer historyDemographics and cancer history

The following patient-reported demographic and clinical characteristics were included: ethnicity, gender, age, insurance status, primary language, cancer type, cancer stage, and status on the cancer care continuum.

#### Pre-intervention barriers assessment

The original PNRP recommended barrier descriptions [30] were modified based on expert consultation (Calhoun) for a total of 88 distinct barriers to cancer care. At the time of enrollment, the community-focused patient navigator conducted a modified PNRP assessment with each patient to identify their barriers to cancer care.

#### Post-intervention barrier resolution definitions and evaluations

The patient navigator utilized a participant-specific barrier tracking log within REDCap to systematically document efforts to resolve each patient-reported barrier to cancer care for each enrolled participant. At the 3-month post-navigation intervention, two trained members of the research team systematically evaluated each participant’s barrier tracking log, reviewed all documented efforts by the navigator, and assessed the extent to which the navigator had resolved each of the participant’s barriers to care. The research team defined levels of barrier resolution in the following ways: (1) “*unaddressed*” indicated that the navigator was not able to identify/provide a satisfactory referral or community resource; (2) “*attempted*” indicated that the navigator worked on a solution and provided a referral or resource for the specific barrier, but full resolution of that patient’s barrier was not documented; (3) “*resolved*” indicated that the navigator provided a resource that resulted in a clearly documented barrier resolution for that patient. Prior to data analysis, a new variable was created, “*addressed*,” which included the combination of “attempted” and “resolved” to represent the broadest effort made by the navigator to, at a minimum, address each barrier. At the barrier level, “*barrier resolved*” rates were calculated as the total number of times each barrier was “*resolved*” divided by the total number of times that barrier was reported across patients; “*barrier addressed*” rates were calculated as the total number of times each barrier was “*addressed*” divided by the total number of times that barrier was reported across patients. At the patient level, the “*resolved*” rate reflected the total number of barriers “*resolved*” for each patient, divided by their total number of pre-intervention barriers; the “*addressed*” rate reflected each patient’s total barriers “*addressed*” divided by their total pre-intervention barriers.

### Statistical analyses

Prior to data analyses, each of the 88 barrier types on the modified PNRP assessment was reviewed and grouped into one of three overarching domains: *Financial*, *Health Access*, and *Psychosocial* (Appendix 7). This categorization was informed by consultation with the relevant literature [12, 32, 34], and finalized through a coding process where the research team discussed each barrier categorization and resolved disagreements through consensus.

All data were collected in REDCap and de-identified prior to export. Data cleaning, statistical analyses, and visualizations were conducted in SPSS and Stata. Descriptive analyses in SPSS identified frequencies of discrete barriers as well as patient-level associations between barrier counts and resolution percentages. Resolution patterns were explored at both the barrier level and patient level using logistic regressions. For Aim 2, at the barrier level, associations between the barrier domain (e.g. *Financial*) and the likelihoods of resolution were estimated using two logistic regression models: (1) In the “initial model,” domains were regressed against resolution status (i.e., “barrier addressed” or “barrier resolved”), (2) In the “full model,” the following controls were added (with reference categories for each variable group identified in brackets []): race and ethnicity (Hispanic, Native American, [all others]); primary language (Spanish, [English], other), gender (women); age (≤ 49, 50–59, [60–69], ≥ 70); primary payer (Medicaid, Medicare, uninsured/underinsured [all others]); cancer type (breast, gastrointestinal, genitourinary, [all others]); stage ([1 or 2], 3 or 4); and status on the cancer care continuum (detection, palliative care, survivorship, [treatment, other]). This full model tests the extent to which the estimated associations in the initial model were mediated by patient-level characteristics. Standard errors were estimated by clustering at the patient level to account for possible heterogeneity across patients. For Aim 3, at the patient level, associations between the barrier domain (e.g., *Financial*) and the likelihoods of barrier resolution were again estimated using two similarly specified logistic regression models: (1) the “initial model” consisted of only the domain combination dummy variables, and (2) the “full model” included the control variables described earlier. At the patient level, these mutually exclusive dummy variables were created to describe all possible barrier domain combinations, which were then regressed against having at least one barrier “addressed” and having at least one barrier “resolved.” To further characterize barrier resolution likelihood, negative binomial regressions were conducted with the total number of barriers reported, per patient, as the exposure factor. In these models (Table 6), the exponents of the regression coefficients are interpreted as incident rate ratios, or the ratio of adjusted resolution rates, and the reference group was participants who endorsed barriers in all three barrier domains.

## Results

### Sample description

A total of 207 patients with cancer completed a 3-month community-focused patient navigation intervention. Descriptive statistics are reported in Table 1. The majority of participants self-identified as Hispanic/Latino (61.8%) and reported Medicaid coverage (32.9%). Approximately half were 60 years of age or older (53.1%). A sizeable minority of patients reported Spanish as their primary language (43.0%) and uninsured/underinsured as their insurance coverage (24.2%). Gastrointestinal cancer was the most common cancer type (25.1%), followed by breast cancer and genitourinary cancer (17.4% each). “Other” cancers included hematological (12.1%), head and neck (10.1%), and skin, lung, and brain (each less than 10%). Half of the participants were in late stage (stage III or IV) disease (driven largely by those with GI cancer), although information on cancer stage was not available for 21.3% of participants. Most participants were recently diagnosed or had recently initiated treatment (49.3%). For a complete description of participant demographic and cancer characteristics, see Ver Hoeve et al., 2024 [8].

**Table 1:** Demographic and cancer history characteristics

	Sample	Hispanic/Latino	Non-Hispanic/Latino White	Other race/ethnicity
***N***	207 (100%)	128 (61.8%)	63 (30.4%)	16 (7.7%)
Gender				
Women	110 (53.1%)	67 (52.3%)	33 (52.4%)	10 (62.5%)
Age				
Age ≤ 49	42 (20.3%)	33 (25.8%)	6 (9.5%)	3 (18.8%)
Age 50–59	55 (26.6%)	36 (28.1%)	16 (25.4%)	3 (18.8%)
Age 60–69	71 (34.3%)	40 (31.3%)	27 (42.9%)	4 (25.0%)
Age ≥ 70	39 (18.8%)	19 (14.8%)	14 (22.2%)	6 (37.5%)
Insurance status				
AHCCCS (Medicaid)	68 (32.9%)	47 (36.7%)	15 (23.8%)	6 (37.5%)
Medicare/federally funded	51 (24.6%)	24 (18.8%)	21 (33.3%)	6 (37.5%)
Private/commercial	38 (18.4%)	19 (14.8%)	15 (23.8%)	4 (25.0%)
Uninsured/underinsured	50 (24.2%)	38 (29.7%)	12 (19.0%)	0 (0.0%)
Primary language				
English	113 (54.6%)	43 (33.6%)	59 (93.7%)	11 (68.8%)
Spanish	89 (43.0%)	85 (66.4%)	2 (3.2%)	2 (12.5%)
Other	5 (2.4%)	0 (0.0%)	2 (3.2%)	3 (18.8%)
Cancer type				
Gastrointestinal	52 (25.1%)	31 (24.2%)	16 (25.4%)	5 (31.3%)
Breast	36 (17.4%)	23 (18.0%)	11 (17.5%)	2 (12.5%)
Genitourinary	36 (17.4%)	23 (18.0%)	9 (14.3%)	4 (25.0%)
Other	82 (39.9%)	51 (39.9)%	27 (42.9%)	5 (31.3%)
Cancer stage				
Early (stage I and stage II)	59 (28.5%)	39 (30.5%)	18 (28.6%)	2 (12.5%)
Late (stage III and stage IV)	104 (50.2%)	60 (46.9%)	32 (50.8%)	12 (75.0%)
Other or unknown	44 (21.3%)	29 (22.7%)	13 (20.6%)	2 (12.5%)
Care continuum				
Detection	61 (29.5%)	40 (31.3%)	19 (30.2%)	2 (12.5%)
Treatment initiation	41 (19.8%)	24 (18.8%)	7 (11.1%)	10 (62.5%)
Treatment	51 (24.6%)	31 (24.2%)	19 (30.2%)	1 (6.3%)
Palliative/survivor/other	54 (26.1%)	33 (25.8%)	18 (28.6%)	3 (18.8%)

Note: “Other” cancer types include hematological, head and neck, skin, lung, and brain.

### Aim 1: Characterization of barriers to cancer care at pre-intervention

At pre-intervention, the average number of discrete barriers endorsed by individual participants was 3.54 (range, 1–10). As previously reported, the number of patient-reported barriers was not significantly associated with participant ethnicity, race, primary language, gender, age, or insurance status (*p*’s > 0.05), nor did the number of barriers differ by cancer type, cancer stage, or status along the cancer care continuum (*p*’s > 0.05) [10]. Across the full sample of 207 participants, a total of 732 barriers were reported. Fifty-two of the 88 potential barriers listed on the assessment were endorsed by at least one participant. The 10 most frequently endorsed pre-intervention barriers to cancer care (Table 2) represented 72% of all reported barriers in this study. The four most endorsed barriers included “can’t afford utilities” (*n* = 85), “needs vision care” (*n* = 78), “can’t afford housing” (*n* = 75), and “public transportation not accessible” (*n* = 56).

**Table 2:** Barrier endorsement (pre-intervention) and resolution (post-intervention)

	Pre-intervention	Post-intervention		
	Count	Unaddressed (%)	Addressed (attempted + resolved) (%)	Resolved (%)
Barriers				
All barriers	732	191 (26.1%)	541 (73.9%)	180 (24.6%)
Top ten barrier types				
Can’t afford utilities	85	21 (24.7%)	64 (75.3%)	10 (11.8%)
Needs vision care	78	5 (6.4%)	73 (93.6%)	32 (41.0%)
Can’t afford housing	75	28 (37.3%)	47 (62.7%)	3 (4.0%)
Public transportation not accessible	56	12 (21.4%)	44 (78.6%)	21 (37.5%)
Limited or no health insurance	48	14 (29.2%)	34 (70.8%)	14 (29.2%)
Can’t afford high copay	48	17 (35.4%)	31 (64.6%)	9 (18.8%)
Does not have PCP	45	10 (22.2%)	35 (77.8%)	8 (17.75%)
Needs hearing test	35	16 (45.7%)	19 (54.3%)	2 (5.7%)
Feels depressed	31	6 (19.4%)	25 (80.6%)	9 (29.0%)
Feels overwhelmed by paperwork	26	4 (15.4%)	22 (84.6%)	10 (38.5%)
Barrier domains^a^				
Health Access domain	336	75 (22.3%)	261 (77.7%)	96 (28.6%)
Financial domain	313	96 (30.7%)	217 (69.3%)	61 (19.5%)
Psychosocial domain	83	20 (24.1%)	63 (75.9%)	23 (27.7%)

Note: The barrier “Addressed” category was calculated as a combination of barriers that were assessed as either “attempted” or “resolved” to represent the broadest definition of efforts made to address a specific barrier

^a^Based on a Pearson chi-square test (df = 2), the variation in resolution rates across the three barrier domains was statistically significant with *p* = 0.021

Of the endorsed barriers (*n* = 52), 21 (40.4%) were categorized within the “*Health Access*” domain, 15 (28.8%) were categorized within the “*Financial*” domain, and 16 (30.8%) were categorized within the “*Psychosocial*” domain (Appendix). “*Health Access*” represented the most frequently endorsed domain (*n* = 336 endorsements), followed closely by “*Financial*” (*n* = 313 endorsements). Within our sample, 82.1% of participants endorsed having at least one barrier within the *Health Access* domain, 73.9% endorsed having at least one barrier within the *Financial* domain, and 33.3% reported having at least one barrier within the *Psychosocial* domain (Table 3). Further examination of the distribution of barriers indicated that 11.6% of participants exclusively reported barrier types within the *Financial* domain, 16.4% exclusively reported barrier types within the *Health Access* domain, and 0.5% exclusively reported barrier types within the *Psychosocial* domain. For this sample of participants, the most commonly endorsed domain combination was *Financial* and *Health Access* (38.6%). A sizeable minority of participants (17.9%) reported barriers within all three domains (Table 3).

**Table 3:** Patient-level barrier endorsement (pre-intervention) and resolution (post-intervention)

Patient-level characterization of barrier domains	Full sample (*n* = 207)
*Pre-intervention*	
Average number of individual barriers per participant	3.54
Barrier domain (*Financial*, *Health Access*, *Psychosocial*) endorsements	
Endorsed ≥ 1 barrier in *Financial*	73.9%
Endorsed ≥ 1 barrier in *Health Access*	82.1%
Endorsed ≥ 1 barrier in *Psychosocial*	33.3%
Barrier domain combination endorsements	
Endorsed ONLY barrier(s) in *Financial*	11.6%
Endorsed ONLY barrier(s) in *Health Access*	16.4%
Endorsed ONLY barrier(s) in *Psychosocial*	0.5%
Endorsed BOTH *Financial* and *Health Access*	38.6%
Endorsed BOTH *Financial* and *Psychosocial*	5.8%
Endorsed BOTH *Psychosocial* and *Health Access*)	9.2%
Endorsed barriers in ALL (*Financial*, *Health Access*, *Psychosocial*)	17.9%
*Post-intervention*	
Average patient-level rate of barriers “Addressed”	75.0%
Average patient-level rate of barriers “Resolved”	25.7%
Patient-level resolutions: barrier domains (*Financial*, *Health Access*, *Psychosocial*)	
Had ≥ 1 barrier(s) “addressed” in *Financial* domain	81.7%
Had ≥ 1 barrier(s) “resolved” in *Financial* domain	32.7%
Had ≥ 1 barrier(s) “addressed” in *Health Access* domain	88.2%
Had ≥ 1 barrier(s) “resolved” in *Health Access* domain	43.5%
Had ≥ 1 barrier(s) “addressed” in *Psychosocial* domain	78.3%
Had ≥ 1 barrier(s) “resolved” in *Psychosocial* domain	31.9%

### Aim 2: Barrier resolution at the barrier and barrier domain levels

Of the 732 endorsed barriers, 26.1% were “*unaddressed*” and 73.9% were “*addressed*” (i.e., “attempted” + “resolved”). Within “*addressed*,” 24.6% of barriers were “*resolved*” at post-intervention analysis (Table 2). Barriers within the *Health Access* domain were “*addressed*” in 77.7% of cases and “*resolved*” in 28.6% of cases. Barriers within the *Financial* domain were “*addressed*” in 69.3% of cases and “*resolved*” in 19.5% of cases. Barriers within the *Psychosocial* domain were “*addressed*” in 75.9% of cases and “*resolved*” in 27.7% of cases.

Among the top 10 most endorsed barriers at pre-intervention, rates of “*addressed*” and “*resolved*” differed descriptively (Table 2). Barriers with the highest “*resolved*” rates were (1) “Needs Vision Care” (*Health Access* domain; resolved in 41.0% of instances), (2) “Feels Overwhelmed by Paperwork” (*Psychosocial* domain; resolved in 38.5% of instances), and (3) “Public Transportation Not Readily Available” (*Health Access* domain; resolved in 37.5% of instances). Notably, some barriers exhibited high barrier “*addressed*” rates, but relatively low barrier “*resolved*” rates (Table 2). For example, the most frequently reported barrier, “can’t afford utilities” had an “*addressed*” rate of 75.3%, but an 11.8% “*resolved*” rate. Similarly, the barrier “can’t afford housing” was “*addressed*” in 62.7% of instances but only “*resolved*” in 4% of instances at post-intervention.

At the barrier domain level, resolution rates differed significantly. As indicated in Table 4, the odds of *Financial* domain barriers being “*addressed*” were 35% lower (OR = 0.65; 95% CI = 0.45, 0.93) relative to barriers in the *Health Access* domain. The initial estimate of difference in “*addressed*” likelihood between domains remained robust, even after controlling for key demographic, socioeconomic, and cancer-related characteristics (OR = 0.63; 95% CI = 0.43, 0.93; *p* < 0.05). Similarly, when looking barrier resolution, the odds of *Financial* barriers being “*resolved*” were 39% lower relative to *Health Access* barriers. And again these results remained robust with the inclusion of control variables (OR = 0.56, 95% CI = 0.37, 0.87).

**Table 4:** Barrier-level logit models

Barrier Addressed				Barrier Resolved				
	Barrier Domain Only		Barrier Domain + Controls		Barrier Domain Only		Barrier Domain + Controls	
N	732		732		732		732	
Mean of dependent variable	0.739		0.74		0.246		0.25	
Pseudo R-squared	0.007		0.039		0.010		0.037	
	**OR**	**95% CI**	**OR**	**95% CI**	**OR**	**95% CI**	**OR**	**95% CI**
Financial domain	0.65*	(0.45, 0.93)	0.63*	(0.43, 0.93)	0.61*	(0.41, 0.89)	0.56**	(0.37, 0.87)
Psychosocial domain	0.91	(0.53, 1.53)	0.96	(0.56, 1.65)	0.96	(0.55, 1.67)	1.00	(0.55, 1.80)
Health access domain (ref)			X				X	
Demographic controls			X				X	
Payer controls			X				X	
Cancer type and stage controls			X				X	
Care continuum controls			X				X	

Note: *corresponds with *p* < 0.05 and ** corresponds with *p* < 0.001. Demographic controls: gender, age, race/ethnicity, primary language; payer controls: Medicaid, Medicare, uninsured/underinsured; cancer type and stage controls: gastrointestinal, breast, genitourinary, all others, late stage (III and IV); care continuum controls: detection, palliative care, survivorship, all other care phases. Standard errors clustered at the patient level.

### Aim 3: Barrier resolution at the patient level

At the patient level, participants had, on average, 75% of their initial barriers “*addressed*” (i.e., “attempted” + ”resolved”), while 25% were “*unaddressed.*” Among “*addressed*” barriers, 25.7% were assessed as “*resolved*” (Table 3). Within the Barrier Domains, 32.7% of participants had at least one of their barriers from the *Financial* domain “*resolved*”; 43.5% had at least one of their barriers from the *Health Access* domain “*resolved*”; and 31.9% had at least one of their barriers from the *Psychosocial* domain “*resolved.*” Resolution rates did not differ significantly by demographic or cancer characteristics (*p* > 0.05).

For participants who reported barriers exclusively within the *Financial* domain, the odds of any of their barriers being “*resolved*” were approximately 77% lower compared to participants who reported barriers in each of the three domains (Table 5). This estimate did not change with the addition of demographic and cancer controls (OR = 0.20; 95% CI = 0.05, 0.72). For participants who reported barriers exclusively within the *Health Access* domain, the odds of any of their barriers being “*addressed*” were significantly lower (86%) compared to participants who reported barriers within each of the three domains (Table 5). Estimates did not change with the addition of demographic and cancer controls (OR = 0.10; 95% CI = 0.02, 0.45). Across the other patient-level combinations of domain barriers for “*addressed*” and “*resolved*,” the odds were not significantly different, and estimates were largely unaffected by the addition of demographic and cancer controls (Table 5).

**Table 5:** Patient-level logit models

	Any barrier addressed				Any barrier resolved			
	Barriers only		Barriers + controls		Barriers only		Barriers + controls	
*N*	207		207		207		207	
Mean of dependent variable	0.83		0.83		0.56		0.56	
Pseudo *R*-squared	0.07		0.19		0.03		0.09	
	OR	95% CI	OR	95% CI	OR	95% CI	OR	95% CI
Financial only	0.33	(0.07, 1.52)	0.31	(0.06, 1.68)	0.23**	(0.08, 0.69)	0.20*	(0.05, 0.72)
Healthcare access only	0.14**	(0.04, 0.55)	0.10**	(0.02, 0.45)	0.46	(0.18, 1.21)	0.47	(0.16, 1.34)
Financial and access	0.60	(0.15, 2.33)	0.69	(0.17, 2.80)	0.66	(0.29, 1.49)	0.64	(0.26, 1.54)
Financial and psychosocial	0.94	(0.09, 10.07)	0.69	(0.06, 7.44)	0.46	(0.12, 1.74)	0.49	(0.12, 1.98)
Access and psychosocial	0.46	(0.08, 2.53)	0.27	(0.04, 1.78)	0.63	(0.20, 1.99)	0.88	(0.24, 3.21)
Barriers in each domain (ref)			X				X	
Demographic controls			X				X	
Payer controls			X				X	
Cancer type and stage controls			X				X	
Care continuum controls			X				X	

Note: * corresponds with *p* < 0.05 and ** corresponds with *p* < 0.001. The first panel shows the results from the patient-level logits estimating the association between having at least one barrier “addressed” and the mutually exclusive combination of domains that characterized the patient’s mix of barriers. Only one patient reported having only *Psychosocial* barriers, and this patient was grouped with those who reported both *Health Access* and *Psychosocial* barriers. Demographic controls: gender, age, race/ethnicity, primary language; payer controls: Medicaid, Medicare, uninsured/underinsured; cancer type and stage controls: gastrointestinal, breast, genitourinary, all others, late stage (III and IV); care continuum controls: detection, palliative care, survivorship, all other care phases. Reference category = barriers in each domain

Finally, we analyzed the relationship between the combinations of barriers across domains and the *number* of barriers addressed and resolved at the patient level, using negative binomial regressions (Table 6). Although none of these results was statistically significant, findings from Table 6 suggest that participants who endorsed only *Financial* barriers had the lowest adjusted barrier-resolution rates of the sample, while those with only *Health Access* barriers had the highest barrier resolution rates (Table 6).

**Table 6:** Patient-level negative binomial models

	Barriers addressed			Barriers resolved			
	Barriers only		Barriers + controls	Barriers + controls			
*N*	207			207		207	
Mean of dependent variable	2.61			0.87		0.87	
Pseudo *R*-squared	0.291			0.960		0.995	
	IRR	95% CI	95% CI	IRR	95% CI	IRR	95% CI
Financial only	1.06	(0.85, 1.31)	(0.94, 1.30)	0.65	(0.34, 1.25)	0.66	(0.31, 1.41)
Healthcare access only	1.14	(0.97, 1.35)	(0.92, 1.25)	1.34	(0.84, 2.15)	1.44	(0.84, 2.47)
Financial and access	1.10	(0.96, 1.26)	(0.94, 1.18)	0.91	(0.63, 1.32)	0.88	(0.60, 1.29)
Financial and psychosocial	1.10	(0.88, 1.38)	(0.85, 1.38)	0.76	(0.38, 1.53)	0.79	(0.41, 1.53)
Access and psychosocial	1.23*	(1.04, 1.46)	(1.04, 1.45)	1.05	(0.61, 1.81)	1.21	(0.67, 2.22)
Barriers in each domain (ref)						X	
Demographic controls						X	
Payer controls						X	
Cancer type and stage controls						X	
Continuum controls						X	

* corresponds with *p* < 0.05. Demographic controls: gender, age, race/ethnicity, primary language; payer controls: Medicaid, Medicare, uninsured/underinsured; cancer type and stage controls: gastrointestinal, breast, genitourinary, all others, late stage (3 and 4); care continuum controls: detection, palliative care, survivorship, all other care phases

## Discussion

Substantial research supports patient navigation as an efficacious, evidence-based intervention that can reduce patients’ barriers to cancer care and, in turn, enhance health equity [11, 18, 33, 38, 39]. The recent decision by the Centers for Medicare and Medicaid Services to allow reimbursement for patient navigation services, including lay navigation, signals the potential for greater uptake of patient navigation programming across diverse oncology settings. The current study provides a foundation for strengthening patient navigation program effectiveness by introducing precise definitions of patients’ barriers to cancer care, systematically classifying patients’ barriers within financial, health access, and psychosocial domains, distinguishing between “addressing” and “resolving” barriers, and analyzing differences in barrier resolution rates at both the barrier level and patient level.

### Interpretation of key findings

In this study, which included primarily Hispanic/Latino patients enrolled on Medicaid, all participants reported *at least one* barrier and averaged 3.5 distinct barriers to cancer care at the time of enrollment. This represents a higher pre-navigation barrier burden than has been reported in previous patient navigation studies [32, 34, 40], indicating a high level of unmet need and that this study was able to reach and enroll the specific patient population that patient navigation programs were originally designed to support [41]. Unlike other studies that found links between barrier counts, patient race, and insurance types [42, 43], demographic and clinical characteristics were not associated with our pre-intervention barrier counts, potentially suggesting greater homogeneity of our sample. That said, demographic and clinical characteristics were also not associated with barrier resolution likelihood at post-intervention, suggesting that resolution likelihood was dependent primarily on the barrier types themselves, as opposed to the demographic or disease characteristics of the sample participants.

Rates of barrier resolution differed significantly between the *Health Access*, *Financial*, and *Psychosocial* barrier domains such that the odds of barrier resolution following community-focused patient navigation were 60% higher for a barrier within the *Health Access* domain compared to the *Financial* domain. This difference remained significant even after controlling for key demographic and disease covariates. At the patient level, the odds of barrier resolution for participants who reported having only barriers in the *Financial* domain were 77% lower compared with participants who reported barriers within each of the three domains. Taken together, these results indicate that barriers within the Financial domain had the lowest odds of being resolved in the context of this community-focused patient-navigation intervention.

Financial burden or financial toxicity has been linked to deleterious cancer health outcomes [44, 45], particularly for patients who experience the greatest levels of unmet need [46]. The fact that our community-focused patient navigation program was least effective at reducing *Financial* barriers is consistent with past literature, and suggests that future community-focused patient navigation programs may need to consider (a) enhancing navigator training regarding financial barrier resolution strategies, or (b) delegating financial barriers to financial navigators, thus allowing community-focused navigation programs to concentrate specifically on the areas of barrier resolution where they are more effective (i.e., resolving *Health Access* and *Psychosocial* barriers) and/or (c) sharing financial barrier data with health system leaders who might impact institutional or political policy. Additionally, future iterations of patient navigation programs should consider the use of digital tools such as AI-based risk stratification and structured information platforms [47] to enhance access to reliable information and decision-making support, particularly for financial barrier challenges. Accumulating intervention science research emphasizes the critical need for continuous intervention refinement [48–50]; our findings support the recommendation for ongoing optimization of patient navigation interventions, especially as it relates to issues of financial burden.

### Study limitations

This paper is not without limitations. A more standardized qualitative system for recording the navigator’s process-level reflections on barrier resolution efforts, in real-time, would have added a meaningful layer of depth to these barrier resolution characterizations. Robust process-level data collection would have shed light on institutional, community, and state-level social service availability, potentially affecting barrier resolution. Additionally, our carefully constructed definitions of “addressed” and “resolved” barriers were developed by this research team, including an active member of PNRP, with a goal of developing future consensus on terminology, but obtaining collaborative input from additional stakeholders prior to conducting our study may have been more comprehensive. Also, this study was conducted at an NCI-designated cancer center, but most patients with cancer in the USA are not treated at NCI-designated cancer centers [51]. Efforts to address cancer health disparities must also consider navigator presence and efforts in community cancer settings. Although our intervention demonstrated implementation effectiveness [8], our analyses did not directly assess how barrier resolution might be associated with patient satisfaction, which could have implications for future optimization of patient navigation programs. Finally, we relied on participants’ intake responses to characterize insurance status; however, collecting insurance status from the electronic health record, as opposed to patient report, may have improved accuracy.

## Conclusions

This paper makes several notable contributions to the literature. First, actionable definitions (i.e., barrier “addressed” vs. barrier “resolved”) allowed for a nuanced assessment of patient-reported barriers and their amenability to resolution via community-focused patient navigation. Tracking of specific barriers over the intervention period followed by systematic assessment of barrier resolution served as a metric for evaluating patient navigation program effectiveness. As a result of a priori definitions, we demonstrated that patient navigation interventions are not equally effective at resolving all barriers to cancer care. For example, our most frequently reported barrier, “can’t afford utilities” had an “addressed” rate of 75.3% but only “resolved” in 11.8% of instances. This suggests that simply providing a useful resource at one time point (i.e., satisfying the criteria for barrier “addressed”) may not lead to the full resolution of that barrier. Another unique contribution of our study was our use of logistic and negative binomial regression to demonstrate that resolution likelihood was dependent primarily on the barriers themselves, as opposed to patient-related factors. Finally, although substantial research on patient navigation—including efficacy studies—has been conducted over the past 30 years, the majority of studies have narrowly focused on patients with only one cancer type (e.g., breast cancer) and at only one point within the cancer care continuum [38]. The diverse demographic and disease makeup of the participants in our study allow for stronger generalization of our findings across the cancer care continuum.

In conclusion, achieving health equity for underserved patients with cancer requires implementation of evidence-based interventions that target the primary drivers of healthcare disparities including health access, financial, and psychosocial barriers. Based on our findings, barriers within the Financial domain had the lowest odds of being resolved in the context of this community-focused patient-navigation intervention. Fine-tuning of barrier-specific resolution effectiveness as outlined in this paper can provide a framework for future optimization of patient navigation interventions with a goal of ultimately ameliorating cancer health disparities.

## Data Availability

The data that support the findings of this study are available from the authorship team upon reasonable request.

## Appendix

**Table 7:** Endorsed barrier types categorized into overarching barrier domains

*Financial* barrier domain	*Health Access* barrier domain	*Psychosocial* barrier domain
• Can’t afford housing	• Physical disability or needs wheelchair	• Doesn’t feel comfortable speaking English
• Can’t afford food	• Needs vision care/has visual impairment	• Staff/providers don’t speak in native language
• Can’t afford utilities	• Has hearing impairment	• No access to interpreter
• Can’t afford clothing	• Has balance issues	• Does not have reliable partner/family
• Can’t afford cancer treatment test or device	• Limited or no health insurance	• In an emotionally abusive home environment
• Can’t afford lodging for overnight stays	• Needs to follow-up with insurance	• Patient worries about emotional support for spouse
• Can’t afford bus/train fare or gas money	• No dental care	• Mental health problems
• Can’t afford high co-pay/deductible	• Has poor nutrition	• Feeling too stressed out
• Can’t afford medications	• Has poor hygiene	• Feeling depressed/unmotivated
• Homeless	• No primary care provider	• Alcohol/substance abuse
• Unstable housing	• Needs help finding specialty care	• Worried will lose job/health benefits
• No sick time/paid time off	• Needs help with medication management	• No family/friend who can give patient ride to clinic
• Doesn’t have own telephone/phone disconnected	• Needs help to stop smoking	• Overwhelmed by paperwork
• House needs cleaning	• Needs help scheduling cancer-related appointments	• Patient does not understand what their insurance covers
• Other Financial (e.g., needs pullout couch, car needs repair, needs storage unit, patient’s roof needs repair)	• Needs help scheduling specialty appointments	• Bills are a stressor/patient worries about getting bills
	• Needs case management and care coordination	• Fear of dying
	• Public transportation is not readily available	
	• Long waits at the clinic and patient could not stay	
	• Patient unaware of eligibility for medical care services	
	• No living will	
	• No medical directive	
